# Animal Models to Study Mucormycosis

**DOI:** 10.3390/jof5020027

**Published:** 2019-03-27

**Authors:** Ilse D. Jacobsen

**Affiliations:** 1 Research Group Microbial Immunology, Leibniz Institute for Natural Product Research and Infection Biology (Hans Knöll Institute), Beutenbergstrasse 11a, 07745 Jena, Germany; ilse.jacobsen@leibniz-hki.de; Tel.: +49-3641-532-1223; 2 Institute of Microbiology, Faculty of Biological Sciences, Friedrich Schiller University Jena, 07743 Jena, Germany

**Keywords:** mucorales, mucormycosis, pathogenesis, infection models, mouse models, *Galleria mellonella*, *Caenorhabditis elegans*

## Abstract

Mucormycosis is a rare but often fatal or debilitating infection caused by a diverse group of fungi. Animal models have been crucial in advancing our knowledge of mechanisms influencing the pathogenesis of mucormycoses, and to evaluate therapeutic strategies. This review describes the animal models established for mucormycosis, summarizes how they have been applied to study mucormycoses, and discusses the advantages and limitations of the different model systems.

## 1. Introduction

The subphylum *Mucoromycotina* within the order Mucorales comprises a diverse group of ancient fungi [1,2,3], of which several are able to cause infections in humans. The most commonly encountered agents in clinical cases belong to the genera *Rhizopus*, *Mucor*, and *Lichtheimia* (formerly *Absidia* and *Mycocladus*). Species of other genera, such as *Rhizomucor*, *Saksenaea*, *Cunninghamella*, and *Apophysomyces*, occasionally cause disease [4,5,6]. Although mucormycosis is rare compared to other fungal infections, the clinical course of disease is often rapidly progressive and associated with a high mortality despite aggressive therapy [7,8,9,10].

Similar to other invasive fungal infections, immunosuppression (especially prolonged and severe neutropenia, malignant hematological disease with or without stem cell transplantation, and the prolonged use of corticosteroids) predisposes to mucormycosis [4,6,10]. Further risk factors include poorly controlled diabetes mellitus with or without diabetic ketoacidosis, iron overload, and therapy with the iron chelator deferoxamine [4,6,10]. The most common route of infection is the inhalation of fungal spores, resulting in pulmonary or rhino-orbital-cerebral forms of mucormycosis. In addition, infections with Mucorales spp. have been observed after a major trauma, including burn wounds [11,12,13,14,15], caused by the direct inoculation of spores into the tissue. In these cases, infections can occur in the absence of other risk factors. In a few cases, spores entered the tissue via minor injuries such as insect bites or animal scratches [11,16,17]. Gastrointestinal mucormycosis has also been reported, especially from Asia, and likely results from ingested spores [4,18,19]. An important feature shared by the different types of mucormycoses is the invasion of blood vessels and subsequent thrombosis leading to tissue necrosis. Angioinvasion also explains the dissemination of infection that is often observed in mucormycosis [20,21,22,23].

The specific histopathological alterations caused by mucormycosis, the rapidly progressive nature of this infection, and the extensive tissue necrosis commonly accompanying it are unique features that differentiate mucormycosis from other mold infections, for example, invasive aspergillosis. Furthermore, diabetes mellitus, iron overload, and deferoxamine therapy are risk factors specific for mucormycosis. These unique aspects also have implications for the use and evaluation of animal models employed to study these devastating infections.

Animal models are essential in understanding infectious diseases and in the development of therapy in general. They are probably even more important for a better understanding of diseases like mucormycoses, for which access to patient material is limited and the design of clinical trials is difficult due to the relatively low incidence, variety of causative agents, different routes of infections, and variation in underlying diseases and risk factors. Various model hosts, ranging from mammalian species such as laboratory mice over other vertebrates to alternative invertebrate hosts, have been employed to analyze pathogenesis and the impact of potential risk factors on infection, to compare the virulence of mucoralean species and strains, and to determine the efficacy of antifungals. The aim of this review is to provide an overview of the different animal models used to study mucormycosis and to discuss the advantages, limitations, and challenges associated with the different models.

## 2. Mammalian and Other Vertebrate Models of Mucormycoses

Mammalian species are, in general, considered to be the gold standard for studying human diseases due to similarities in anatomy and physiology. The suitability of a model host for studying infectious diseases, furthermore, depends on its susceptibility to the infectious agent; ideally, disease in the animal model should reflect the pathological alterations observed in human patients. Clinical cases of mucormycosis featuring the typical hallmarks of human disease have been described in different mammalian species and in birds [24,25], indicating a broad host range of the fungi and conserved pathogenesis. Indeed, a variety of species, ranging from laboratory mice, rats, guinea pigs, and rabbits, to more exotic species like bank voles or Asian water buffalo calves can be infected experimentally with pathogenic mucormycetes [25,26]. Most studies, however, used mice or rabbits [26], and the overview of the published studies presented in Table 1, Table 2 and Table 3 focuses on these two host-species. In addition to different host species, various routes of infection can be used to model the different types of mucormycosis, and different protocols are used to establish the risk factors. The following sections briefly describe the models used for different types of mucormycosis and how the predisposing factors can be incorporated into the models.

### 2.1. Routes of Infection Used to Model Different Types of Mucormycosis

#### 2.1.1. Pulmonary Mucormycosis

The most common route of infection in humans is the inhalation of spores. To mimic this route, the introduction of spores into the airways of laboratory animals can be achieved in two ways: (i) exposure to wet or dry aerosols containing fungal spores or (ii) the direct application of a spore solution onto the nostrils (intranasal) or into the trachea (intratracheal/endotracheal). The use of different aerosolization systems has been described for *Aspergillus* models and likely best reflects the natural mode of infection [27,28,29]; this method has, however, not yet been used to study mucormycosis, likely due to the technical requirements and efforts necessary for standardization. Instead, pulmonary mucormycosis is commonly established by the intranasal or intratracheal/endotracheal application of spores (Table 1). Intranasal application is noninvasive, whereas traditional methods of intratracheal instillation in small rodents involved surgical procedures to gain access to the trachea. Novel techniques, however, facilitate noninvasive intratracheal application [30,31,32], making this technique more attractive. The two methods differ in where the spores are deposited: Following intranasal application, the spores need to pass the upper airways to reach the lung and might attach to the nasal respiratory epithelium [33]. This is circumvented by the intratracheal application, which might, however, lead to a more focal deposition in the lung [34]. Whether the technique used for application influences the subsequent development of mucormycosis has not been systematically investigated, but both the intranasal and intratracheal applications were used in recent publications (Table 1). It should be noted that both approaches require some training and experience to achieve reliable results; sacrificing some animals shortly after infection to determine the spore number in pulmonary tissue can be useful to ensure the reproducibility and to determine the effective infectious dose [35,36].

Usually, infection is performed with resting spores; immunocompetent, healthy animals clear these spores within days to weeks without developing clinical disease [35,37]. It should, however, be noted that a recent publication showed that an infection with germinating spores led to a lethal infection also in immunocompetent animals, likely because the swollen conidia were less phagocytosed [38]. In the presence of risk factors such as immunosuppression or ketoacidosis (see below), fungal growth is insufficiently restricted, resulting in pulmonary lesions. These lesions are histologically characterized by angioinvasion, intravascular thrombosis, coagulative necrosis, and pulmonary hemorrhage [26]. Thus, experimental pulmonary mucormycosis in mammalian models closely resembles the hallmarks of infection in humans.

#### 2.1.2. Disseminated Mucormycosis

In humans, disseminated mucormycosis usually originates from a local infection focus, commonly the lung, and most often occurs in patients with hematological malignancies which are severely immunocompromised [4,6,7,23]. Dissemination to internal organs, including the brain, has also been described following experimental pulmonary mucormycosis in immunocompromised rabbits [64] and mice [35,49], as well as in ketoacidotic mice [49]; most studies using pulmonary models, however, focused their analysis on the lung, and it, thus, remains unclear (i) how frequently and reproducibly dissemination occurs in mammalian models, (ii) if differences between mammalian species exist, (iii) if dissemination rates are different for different mucoralean species and strains, and (iv) whether distinct internal organs are preferentially targeted. Furthermore, dissemination after pulmonary infection has been shown to vary significantly between individual animals and appears to require that the animals survive the acute pulmonary infection [35], which makes the development of standardized models of disseminated mucormycosis via pulmonary infection difficult.

To circumvent these problems, fungal spores can be injected directly into the blood stream (intravenously), leading to the primary dissemination into various organs such as the kidneys and brain. This approach is easy to standardize and leads to a lethal disease even in animals without underlying risk factors [67,68,69,70,71,72]. It should, however, be noted that this approach differs from most human cases in that (i) it circumvents the establishment of primary infections in other organs, and (ii) spores rather than hyphal fragments are reaching the blood stream. This might have an impact on the pathophysiology, the contribution of fungal factors to disease, and the comparative virulence of species and strains. Despite these limitations, an intravenous infection has been shown to be useful to investigate the aspects of pathogenesis for *Mucor* [61,73] and Rhizopus [74,75,76,77] and the efficacy of therapeutic approaches (see Section 2.3).

#### 2.1.3. Other Forms of Mucormycosis

Rhino-orbital mucormycosis in humans originates in the paranasal sinuses, affects the soft tissue of the nose and sinuses, can lead to bone destruction, and can ultimately spread to the brain (rhino-orbital-cerebral disease). Although this form of mucormycosis is relatively common in humans, comprising 20%–39% [7,10,20] of all mucormycosis patients, and is the most common form in cases with diabetes mellitus as underlying risk factor [7,10], it has rarely been studied in animals. The main reason for this is the difficulty in establishing a localized infection: A local intranasal application without the dispersal of spores to either the lung (in anaesthetized animals) or the gastrointestinal tract (by swallowing) is nearly impossible to achieve. Even though the direct intraethmoidal application of spores is possible and has been shown to lead to rhino-cerebral mucormycosis in mice with diabetes mellitus [78,79], dissemination to the lung and other organs occurs in this model [79]. The direct intracerebral injection of spores induces a lethal infection of the central nervous system in immunocompetent C3H mice [70], but this route of infection is not routinely used.

Similar to rhino-orbital-cerebral mucormycosis, cutaneous/subcutaneous infections have rarely been studied experimentally. A localized subcutaneous infection by *Rhizopus* can be established in diabetic rabbits and rats and in immunocompromised mice by the subcutaneous injection of spores [80,81,82,83]. Furthermore, the intradermal injection of distinct *Rhizomucor* strains and *Lichtheimia* spp. can induce transient lesions in immunocompetent mice [69,84,85]. In rabbits, subcutaneous infections leads to granuloma formation [80,81]; interestingly, the fungi persist within these granulomas, and infection can be reactivated by the induction of diabetic ketoacidosis [80]. Even though these models might not reflect the more complex pathophysiological alterations associated with traumatic skin injuries and the subsequent infections, they have the advantage of being technically straightforward, and it should, thus, be possible to easily adopt them for future studies.

Finally, gastrointestinal mucormycosis, a rare manifestation of these infections in humans, has so far not been successfully modeled in animals—the oral inoculation of mice with spores did not result in clinical disease [72].

### 2.2. Predisposing Factors: Manipulation of Mammalian Hosts to Mimick Risk Factors in Humans

With the exception of an intracerebral injection or intravenous infection with a sufficiently high dose, lethal mucormycosis in laboratory animals requires the presence of a predisposing factor, similar to the situation in humans. As the two main groups of patients at risk are immunocompromised and diabetic patients with ketoacidosis, these two underlying conditions are commonly induced in laboratory animals to model these two main patient groups. The two following sections describe the principles of how immunosuppression and ketoacidotic diabetes mellitus can be induced; detailed descriptions of the methods can be found in the publications cited in Table 2.

#### 2.2.1. Immunosuppression

Hematological malignancies themselves and the respective treatment, like the preconditioning of stem cell transplantation, are usually associated with severe immunosuppression. Reduced numbers of white blood cells (leukopenia) and especially a reduction of neutrophilic granulocytes (neutropenia) renders such patients highly susceptible to infections by bacterial and fungal pathogens [86]. Thus, leukopenia/neutropenia is a general risk factor for infectious diseases, and various protocols have been established to induce leukopenia/neutropenia in animal models. In mammalian models of mucormycosis, immunosuppressive regimens have largely been adapted from other infection models, for example, invasive aspergillosis [26]. Leukopenia including neutropenia can be readily induced by the application of cytostatic drugs (Table 2), such as cyclophosphamide in mice or cytarabin in rabbits, which target replicating cells including those in the bone marrow. These substances lead to a rapid drop of neutrophil numbers, as the short half-life of these immune cells requires constant replenishment from the bone marrow. In contrast, resident immune cells, such as tissue macrophages, are not acutely affected by cytostatic therapy, and therefore, commonly used protocols additionally apply corticosteroids (for example, cortisone acetate) to impair the function of these cells. With the established protocols (see the references in Table 2), profound immunosuppression indicated by leukopenia and neutropenia can usually be induced within 2–4 days; following infection, leukopenia can be sustained by the repeated application of the cytostatic drug, if necessary. Successful immunosuppression can be easily confirmed by the determination of leukocyte numbers in blood smears or by using hematology counters.

As an alternative to cytostatic therapy, monoclonal antibodies allow the depletion of distinct types of immune cells. Therefore, animals can be rendered temporarily neutropenic without necessarily affecting other immune cell populations [87]. This approach has been used to study the relative impact of distinct immune cell populations on fungal infections, such as aspergillosis and candidiasis [87,88], but has not yet been applied to models of mucormycosis. Interestingly, the mode of neutrophil depletion has been shown to affect the susceptibility of mice to aspergillosis [88]; as various immunosuppressive regimens are used to condition human patients for stem cell transplantation, it might be interesting to investigate whether the risk for mucormycosis is affected by the leukoablative regimen.

In contrast to cytostatic agents, the application of corticosteroids does not reduce the number of circulating neutrophils but impairs their antifungal function [89]. In consequence, patients or animals receiving corticosteroid therapy are susceptible to infections with filamentous fungi, including Mucorales [4], but pathogenesis differs from leukopenic hosts. This has been nicely demonstrated for murine pulmonary aspergillosis, in which fulminant fungal growth, angioinvasion, and tissue necrosis drive pathogenesis in leukopenic mice, while overt neutrophil influx leads to tissue destruction in corticosteroid-treated animals [90]. Furthermore, fungal virulence factors that negatively affect immune cells, like *Aspergillus fumigatus* gliotoxin, play a role in the infection of corticosteroid-treated but not leukopenic animals [91]. Corticosteroid-based immunosuppression protocols established for murine invasive aspergillosis have also been used successfully to study pulmonary mucormycosis (Table 2). As this model mimics mainly patients receiving a prolonged high-dose corticosteroid treatment after solid organ transplantation or as therapy for autoimmune disorders, it complements the leukopenic model by addressing a second important group of patients at risk [6,7,92].

Independent of the mode of immunosuppression, precautions should be taken to prevent spontaneous infections in immunocompromised animals. Depending on the hygiene level of the animal facility, this might include prophylactic treatment with antibiotics. Furthermore, noninfected immunocompromised control groups should be included as “sentinel animals” to detect spontaneous infections and to control for the effect of immunosuppressive therapy on overall animal health, clinical parameters, and, if applicable, immune reactions and possible side effects of therapeutics.

#### 2.2.2. Diabetes Mellitus

Poorly controlled diabetes mellitus associated with ketoacidosis (diabetic ketoacidosis: DKA) is a risk factor for various infections but especially prominent in mucormycosis, as in some epidemiological surveys, it was identified as a predisposing factor in over one third of the mucormycosis cases [7]. Various animal models have been developed in mice and rats to study type 1 and type 2 diabetes: The approaches range from the chemical ablation of insulin-producing cells, over dedicated models with increased rates of spontaneous autoimmune diabetes, to models of obesity-associated insulin resistance and beta cell failure [109,110]. For infection research including animal models of mucormycosis, the chemical ablation of insulin producing beta cells by the application of streptozotocin or alloxan is most commonly used (Table 2). These inducible models have the advantage that they are independent of the genetic background and age of the animals. A single injection of streptozotocin is sufficient for DKA induction, but it is important to use the correct preparation and dosing of the chemical to achieve consistent outcomes without inducing lethality [110,111]. As not all animals may develop ketoacidosis after induction, blood glucose should be measured to confirm hyperglycemia; additionally, ketosis can be determined in urine samples [111]. As with immunocompromised models, the increased general risk of infections needs to be taken into account and prophylactic treatment with antibiotics might be required.

Of note, some studies in mice used combined streptozotocin-induced DKA and moderate immunosuppression with corticosteroids [48]. To which extent the application of corticosteroids to DKA mice affects susceptibility to mucormycosis has not been systematically investigated, but additional immunosuppression might be necessary to render DKA mice susceptible to some mucormycetes, such as *Lichtheimia corymbifera* [35].

### 2.3. Mix and Match—How to Choose Host Species, Route of Infection, and Predisposing Factors

Ideally, an infection model closely mimics the infection in the species of interest, e.g., the human. For mucormycosis, this is generally the case for all common laboratory animal species (reviewed in Reference [26]), and as mentioned above, early studies in animals indeed used a variety of mammalian hosts. However, the majority of studies in the last two decades were conducted on mice (Table 1 and Table 3). This can be explained by the relatively low cost, fast generation time, ease of handling, and availability of research facilities for this species. Mice have the additional advantage that different protocols for immunosuppression and diabetes mellitus are well-established, a large number of tools for molecular analysis of host responses is available, and genetically modified animals can be used to address specific research questions. Rabbits have also been used for studies and, due to their larger size, have some general advantages: It is, for example, possible to perform repeated sampling, and the sample size is larger. The significantly higher costs and space requirements are, however, a disadvantage.

Within a host species, the choice of predisposing factors and routes of infection largely depends on the research question; when deciding on which model to use, it should be taken into account that the mechanisms by which different predisposing factors increase susceptibility to mucormycosis are fundamentally different. Immunosuppression generally lowers the host defense system, resulting in reduced phagocytosis and/or the inactivation of fungal spores. However, as mentioned in Section 2.2.1, pathogenesis in neutropenic hosts is driven by fungal growth while an influx of immune cells contributes to tissue damage in corticosteroid-treated mice. The mechanisms underlying the increased susceptibility of DKA mice differs from both types of immunosuppression: Pathophysiological relevant changes during DKA include elevated extracellular iron levels and the glucose/acidosis-induced enhanced expression of host cell proteins exploited by *Mucorales* for invasion [112]. Thus, predisposing factors are not interchangeable but significantly affect cellular and molecular pathogenesis. Consequently, understanding the differences between the models is essential for using the most suitable model to address specific research questions. This is nicely exemplified by a study by Gebremariam et al. [44], in which the authors specifically induced acidosis and reversed acidosis by a sodium bicarbonate application to demonstrate the specific role of acidosis in the pathogenesis of mucormycosis in DKA mice.

Similarly, the route of infection defines the main organ affected by the fungi and the type and impact of interaction with the host cells. Following pulmonary infection, the first steps of interactions occur with lung epithelial cells, while the lung is not a primary target after intravenous infection [67]. This needs to be considered when translating findings from in vitro approaches to in vivo models. A good example for a consistent approach is the recent paper by Watkins et al., in which the authors identified enhanced epidermal growth factor receptor (EGFR) signaling in vivo in a pulmonary model. They then tested the relevance of EGFR for the interaction of *Rhizopus* and human alveolar epithelial cells in vitro and, based on these results, tested the effect of EGFR signaling inhibition in a pulmonary mouse model [39].

As the pulmonary model is generally considered to account more accurately for the different infection steps in most cases of human mucormycosis, it is not surprising that this model is commonly used to investigate the pathogenesis mechanisms and virulence factors (Table 3). One exception is mucormycosis caused by *Mucor* spp., which has been studied in systemic infection models. An intravenous infection is also commonly used in studies evaluating the efficacy of therapeutic interventions, especially antifungal drugs (Table 3). For these studies, it is important to be able to reliably induce severe infection; furthermore, antifungal should be ideally able to rescue patients from the most severe forms of disease and in the absence of a protective host response. This can be modelled by the intravenous inoculation of immunocompromised mice; if therapeutic interventions are effective in this model, they might also provide a benefit in less severe cases of infection.

### 2.4. Challenges and Limitations of Mammalian Models

Without a doubt, mammalian models, especially the numerous studies in mice, have significantly contributed to our understanding of molecular aspects of mucormycosis, leading to important therapeutic approaches, such as specific iron chelation therapy [112]. They have, furthermore, been essential models for studying the efficacy of antifungal treatment, including combination therapies (Table 3). However, the technical details vary between studies, even within similar model set ups. One example is pulmonary mucormycosis in neutropenic mice, for which the studies differ in the mouse strain used, gender, dosing of immunomodulatory drugs, and spore application techniques (compare, for example, References [39] and [52]). This lack of standardization does not impair the quality of individual studies, but it makes a direct comparison of different studies difficult. It should be mentioned that the limited standardization is not only a challenge for mucormycosis research but a general issue for animal models, for example, models for invasive aspergillosis (reviewed in References [115,116]). Here, some practical aspects associated with the lack of definite standards will be briefly discussed.

- Mouse strain: Due to the lack of comparative studies, it is as of yet unknown whether the genetic background in inbred mouse strains affects the susceptibility to mucormycosis, pathogenesis, or treatment efficacy. From a practical perspective, there is currently no clear evidence that strain or gender have a significant effect on mucormycosis in mice, but these parameters should be reported in publications.
- Dosing and timing of immunosuppressive treatment: While following common principles, individual protocols differ in detail, including the use of pure chemicals versus medical formulations. In addition to reporting sufficiently detailed information in publications, the confirmation of immunosuppression should be performed to ensure the efficacy of the regimen; the indication of leukocyte numbers could be useful to allow a comparison of the protocols between labs.
- Another important aspect of animal models are the readout parameters, which have been discussed in detail in a previous review of animal models for mucormycosis [26] and in the context of aspergillosis [115,116]. Mortality was traditionally used as the main endpoint, but within the concept of the 3Rs [117], regulations in most countries require that animals are euthanized when moribund. In this context, it is important that the objective criteria for moribundancy (humane endpoints) are described with sufficient detail in publications and that data on clinical parameters such as body weight, overall appearance, and behavior are presented. Similarly, the extent of the post mortem evaluation (or presentation of findings thereof) differs between studies; the documentation of macroscopical findings, histopathology, and fungal burden are, without doubt, valuable parameters to more comprehensively describe and evaluate infections, but a definition of the minimal requirements and technical gold standards is still lacking in the field.
- Another aspect of standardization of infection models, in general, are the questions “Which microbial strain should be used?” and “How should microbes be cultured to obtain the inoculum for infection?”. These questions are especially difficult to answer for mucormycosis as (i) different species from different genera are relevant, (ii) strain-specific differences in virulence have been described [35,108], and (iii) it is as of yet unknown whether strain-specific differences depending on the route of infection and predisposing factors exist.

As outlined in Section 1, mucormycosis can affect different anatomical localizations. While pulmonary models and artificial dissemination via the intravenous route are well-established models, reliable and well-characterized models for rhino-cerebral, cutaneous, (burn) wound-associated, and gastrointestinal infections are lacking. Some of these models might be developed based on existing models, for example, for burn wound infections models [118,119,120,121]. Other clinical questions might require the development of completely new models. One example is rhino-orbital mucormycosis, a form of mucormycosis with high mortality rates, for which a combination of extensive surgical debridement and antifungals is recommended [122]. Due to their small size, mice (and likely other rodents) are not ideal model hosts to address this question; larger animals, such as dogs, pigs, or ruminants are likely better suited because their larger size might allow for (i) more time for the development of invasive disease (thereby facilitating the timing of treatment according to disease severity) and (ii) a state-of-the-art surgical intervention. However, the resources required to develop such models regarding costs and infrastructure are immense compared to the use of conventional laboratory rodents; additionally, an interdisciplinary approach involving medical doctors and veterinarians would be necessary.

Furthermore, some aspects of pathogenesis that differ between Mucormycetes and other pathogenic molds, such as *A. fumigatus*, are not yet addressed by specific animal models: Resting spores of pathogenic mucormycetes are readily phagocytosed by macrophages in vitro and in vivo, which prevents germination but does not lead to fungal killing [38,123,124,125]. The intracellular persistence of spores was also observed in the tissues of human patients [38], and it appears possible that this leads to a latent stage that can be reactivated by immunosuppression. Fungal persistence can also occur within granulomas which are formed in rabbit models of both subcutaneous and pulmonary infection [80,126] and in zebrafish [127]. In these granulomas, the fungus remains viable and mucormycosis can be reactivated by inducing diabetic ketoacidosis in previously infected rabbits [80]. This pioneering work from the 1950s provides a clear proof-of-concept for dormant or latent mucormycete infections that was only recently being followed up [127,128].

Finally, all mammalian models are restricted by ethical considerations—the use of mammals in infection models need to be justified and is subject to institutional and national regulations. This restricts the use of mammalian models for certain questions, such as a large-scale investigation of strain-specific differences in virulence or the screening of antifungal compounds. In consequence, alternative infection models have been employed to address questions that require a large number of animals.

## 3. Alternative Model Hosts

While mammalian models have the advantage to share most physiological, anatomical, and immunological properties with humans, ethical and practical considerations limit their application for large-scale screening purposes and strain comparisons. Furthermore, using mammalian models requires specialized facilities and staff, which might not be available to all researchers. Therefore, alternative model hosts that allow infection studies with less ethical and practical constraints have been developed.

As alternative vertebrate models, zebrafish and embryonated chicken eggs have been used in mucormycosis research. Fertilized chicken eggs produced for poultry farming are relatively inexpensive, easy to maintain at 37°C, and can be infected by different routes, including the chorioallantoic membrane, which shares some features with the mammalian lung [129,130]. They have been employed for high-throughput screening to compare the virulence of mucormycetes both on the species and strain level [131,132], and the relative virulence of some strains has been confirmed in murine models [35]. Working with zebrafish requires specialized facilities, but this host has the advantage of being genetically modifiable (reviewed in References [133,134]). Furthermore, zebrafish larvae are transparent and, thus, highly suitable to following the behavior of fungi and immune cells by intravital microscopy [133,135]. This facilitated the analysis of the fish’s host immune response to *Mucor circinelloides*, which depends on the recruitment of phagocytes and the inhibition of spore germination [127,136], as well as granuloma formation at later stages [127]. The recruitment of neutrophils and macrophages also occurs in adult zebrafish, in which *Mucor circinelloides* induces apoptosis of macrophages [137].

Compared to alternative vertebrate hosts, invertebrates are more commonly and increasingly used to study fungal infections. These model hosts have been recently reviewed in detail [138,139], so the following subsections will only briefly introduce the principles and focus on how they have been applied to study infections with Mucorales. The subsections are dedicated to *Galleria mellonella* and *Drosophila melanogaster* only, as *Caenorhabditis elegans* as an alternative invertebrate infection model for fungi [138,139] has not yet been used for mucormycosis.

### 3.1. Galleria mellonella

Larvae of the greater wax moth *Galleria mellonella* are traditionally bred as bait for fishing but, over the last decade, have gained significant importance as a model host to study fungal infections [140,141,142]. *G. mellonella* larvae are inexpensive to purchase and easy to keep and handle, and importantly, they can be kept at 37 °C, even though the maintenance outside of experiments is usually performed at lower temperatures (15–30 °C) [138,143,144]. Infection and application of substances is usually achieved by injection of defined volumes into the hind pro legs [141,143], from where microbes and substances enter the hemolymph and thereby reach the larval tissues [138]. The insect immune system, including that at the larval stage, contains several innate immunity pathways that are conserved in mammals (recently reviewed in detail [145]). Effector functions are mainly mediated by the six different types of hemocytes, which phagocytose microbes and contribute to encapsulation in tissue, antimicrobial peptides, and melaninization [145,146,147,148]. In addition to larval survival, the quantification of fungal burden, histopathology, and the analysis of immune parameters can be performed to characterize the infection process (reviewed in References [140,141,145,148]).

*G. mellonella* infection models, in general, have been applied to compare the virulence of fungal species and strains, to analyze possible virulence traits, and to evaluate antifungal treatment [140]. Larvae were likewise used for these purposes in mucormycetes studies, although the overall number of publications is still low. Four studies compared the virulence of mucoralean species and strains in *G. mellonella*; Maurer et al. found *Rhizopus* spp. to be more virulent than *Rhizomucor* spp. or *Lichtheimia* spp. at both 37 °C and 30 °C, while the relative virulence of *Rhizomucor* spp. and *Lichtheimia* spp. was temperature dependent [149]. Kaerger et al. came to a similar conclusion by comparing the *Rhizopus* species with different thermotolerance profiles in *G. mellonella* at 30°C and embryonated chicken eggs at 37 °C [131]. Using four to six isolates per species, Maurer et al. concluded that the virulence potential was largely linked to the species rather than influenced by strain differences, although individual strains with altered virulence potentials were identified; this is consistent with results obtained for 20 strains of *Rhizopus microsporus* by Kaerger et al. [131,149] but differs from the observations for *Mucor* [108]. These three studies, furthermore, showed a correlation of growth speed and/or spore size with virulence in this model. Additional factors that seemed to influence virulence in *Galleria* were oxidative stress tolerance and spore size [149], while osmotic and cell wall stress resistance did not correlate with the survival of infected larvae [131]. These findings suggest that oxidative stress is a relevant defense mechanism against Mucorales in *Galleria*, which would be consistent with the use of reactive oxygen and nitrogen species as defense mechanisms by model insects [147]. Furthermore, the iron concentration in the growth medium affects virulence [149]. A fourth study used *Galleria* to analyze the virulence potential of a *Mucor* outbreak strain [72]. In this study, several *Mucor* strains were analyzed both in a systemic murine infection model and in *Galleria*, with some differences in the relative strain virulence between these models [72]. That the *Galleria* model is suitable to test whether a distinct gene affects virulence has been demonstrated by Trieu et al. [93]. In this study, 26 siRNA-based mutants were screened for virulence alterations, identifying three mutants with significantly reduced virulence in larvae. This corresponded to two silenced genes; mutants for both these genes displayed severe growth impairments and were also less virulent in mice. The efficacy of antifungal drugs against mucormycosis has been studied in *G. mellonella*, both using established antimycotics [149,150] and exploring the therapeutic effects of rapamycin against Mucorales [151]. All three studies demonstrated interesting findings and compared the in vivo efficacy with the in vitro effects.

### 3.2. Drosophila melanogaster

The fruit fly *Drosophila melanogaster* is a model organism that has been widely used in various fields of biology, including immunology. Its genetics are well-studied, and the availability of mutant collections make it an attractive tool that led, for example, to the discovery of Toll as receptor for immune signaling essential in fungal infections [152,153]. It has been used as a mini-host for various viral, bacterial, and fungal pathogens, including Mucorales [154,155]. In comparison to the other insect host, *G. mellonella*, it has the advantage of genetic manipulation and the availability of molecular tools and probes; it, however, has to be kept at temperatures below 30 °C, and experiments are usually performed at 29 °C [156]. It is furthermore significantly smaller: The adult female flies commonly used for experiments are only 3 mm long, which makes the injection of defined volumes impossible. Instead, the infection is achieved by pricking the dorsal thorax of CO_2_-anesthetized flies with a needle dipped in a suspension containing a defined number of microbes [154,157]. Alternative routes of infection are an external exposure by rolling flies in spores or providing microbes in the food source [158]; these have, however, not been employed to study mucormycosis. Compared to *Galleria*, the use of *Drosophila* requires more specialized equipment and experience in working with the species [159].

In contrast to other fungi that produce infections only in immunocompromised flies, for example, mutants in the Toll pathway [154,157,159], different Mucorales species cause lethal diseases also in wildtype flies [160]. However, immunosuppression, by genetically interfering with fungal recognition or by chemically interfering by the application of corticosteroids, increases the susceptibility of *Drosophila* to mucormycosis [160]. Furthermore, the importance of iron acquisition for mucormycosis appears to be conserved between mammals and flies [160,161]. Glucose levels and nutrition additionally affect the general infection susceptibility [162,163]. *Drosophila* has so far not been used to perform a large-scale comparison of the virulence of different mucoralean species and strains, but the model was employed to determine the relative virulence of distinct strains [77,164]. Furthermore, experiments in the fly model showed that pre-exposure to certain azoles enhances the virulence of mucormycetes [60,165,166], which has been confirmed for voriconazole in mice [60]. Flies were, furthermore, used to test novel antifungal strategies [52,83,167,168]; only two of these studies also performed an analysis in mice, with one confirming the results in mice [52], while the outcome differed in the other study [83].

### 3.3. Challenges and Limitations of Invertebrate Models

As discussed above, invertebrates are used increasingly as convenient alternative hosts for infection studies of mucoralean fungi. The use of *Drosophila* is, however, limited by the need for specialized equipment and rooms to ensure that the adult flies are contained within the facility. This problem can be circumvented with *G. mellonella*, if larvae are purchased and animals are used before pupation. The disadvantage of purchasing larvae, especially those traded as fish bait or food for exotic animals, however, is that the genetic background is not defined and the age and quality of the larvae might vary, which in turns affects survival after infection (reviewed in Reference [144]). This can be avoided by using commercially available standardized larvae specifically bred for scientific purposes [144]. Standardization is also important to facilitate a comparison of different studies [141,143,144,169,170]; as discussed for mammalian models, the definition and use of reference strains, as well as standard techniques are not yet fully established. Further points to consider when choosing between *Drosophila* and *Galleria* are the temperature used for experiments, the precision of the infectious dose, the ability to isolate phagocytes, and the genetic tractability (reviewed in Reference [159]).

The main challenge and possible limitation of invertebrate hosts is, however, the question if and, to which extent, results obtained in these models are transferable to mammals and, ultimately, humans. The lack of an adaptive immune system and specific organs, such as the lung, could affect pathogenesis, even though several other aspects are conserved [141]. A direct comparison of the relative virulence of mucoralean fungi in invertebrate hosts and mammalian models has only been performed in a few studies and with a limited number of strains. One study showed a good correlation [93], while the other found differences [72]. For other fungal species that are more commonly studied, the correlation between murine and insect models appears to be overall good, but there are also notable exceptions (reviewed in Reference [159]). Whether findings can be transferred from one host to another might depend upon the type of feature studied: The ability to grow within a host is essential for pathogenesis, and it, thus, can be expected that strains with severe growth defects under diverse in vitro conditions display reduced virulence in a variety of infection models. Another example is the ability of fungal strains to acquire essential nutrients like iron; iron sequestration as a way to restrict microbial growth occurs not only in vertebrates [171] and insects [172,173] but also in plants [174]. Therefore, it is not surprising that iron acquisition is important for fungal virulence and that iron overload is a risk factor for mucormycosis in both vertebrate and invertebrate infection models [112,149,160]. In contrast, more specific virulence factors might only be relevant in certain conditions; an example is the previously mentioned *Aspergillus fumigatus* gliotoxin that functions as a virulence factor in corticosteroid-treated but not leukopenic mice [91]. In such cases, the use of different infection models is essential to work out the exact function. Thus, while invertebrate models are extremely useful for the initial virulence determination or comparison of a larger number of strains, the results ultimately have to be validated in one or even several mammalian models.

A similar case can be made for the use of invertebrates in pharmacology studies. As demonstrated in the studies reviewed above, insect hosts are useful for assessing the efficacy of innovative therapeutic approaches and the combination of antifungal drugs. However, differences in the host physiology will affect various pharmacological parameters (e.g., pharmacodynamics, pharmacokinetics, and drug metabolism), and thus, strategies successful in invertebrates need to be validated in mammalian models.

## 4. Conclusions

As summarized in this review, different infection models have been established to study pathogenesis, virulence factors, and therapeutic interventions of mucormycosis. All models have advantages and disadvantages (summarized in Table 4), both from practical and technical perspectives and from how accurately they mimic the different manifestation of mucormycosis in humans. In order to choose the right model, it is important to understand the physiology of the model host, the risk factors associated with mucormycosis and if/how they can be modeled, and the specific pathophysiological features of the chosen model.

## Figures and Tables

**Table 1:** The selected studies using pulmonary models of mucormycosis ^1^.

Host Species	Route of Infection	Fungi	References
Mouse	intratracheal	*Rhizopus* spp.	[38,39,40,41,42,43,44,45,46,47,48,49,50,51]
		*Mucor* spp.	[40,43]
		*Lichtheimia* spp.	[40]
		*Cunninghamella* spp.	[40]
	intranasal	*Rhizopus* spp.	[36,52,53,54,55,56,57,58,59,60]
		*Mucor* spp.	[61]
		*Lichtheimia* spp.	[35]
Rabbit	endotracheal	*Rhizopus* spp.	[37,62,63]
		*Mucor* spp.	[37]
		*Cunninghamella* spp.	[37]
	intranasal	*Rhizopus* spp.	[64]
		*Lichtheimia* spp.	[65,66]

^1^ Only studies published after 2000 and available online were included. A comprehensive list of the studies using mammalian species published before 2000 is presented in Reference [26].

**Table 2:** The predisposing factors for mammalian mucormycosis models.

Host Species	Risk Factor	Method	References
Mouse	Immunosuppression	Cyclophosphamide (+/− cortisone acetate or 5-fluorouracil)	[36,39,40,41,42,43,45,47,49,50,51,52,54,55,56,59,61,73,75,83,93,94,95,96,97,98,99]
		Cortisone acetate	[54,57,58,60,61,100]
	Diabetic ketoacidosis	Streptozotocin	[36,44,46,48,53,71,72,74,76,77,79,98,101,102,103,104,105,106,107,108]
Rabbit	Immunosuppression	Cytarabine i.v.	[62]
		Cytarabine + methyprednisolone	[37,63]
		Cyclophosphamide	[66]
		Methyprednisolone	[66]
	Diabetic ketoacidosis	Alloxan i.v.	[64]

**Table 3:** The application of animal models in mucormycosis research ^1^.

Purpose	Host Species	Fungus	Route of Infection	References
Pathogenesis, virulence traits, comparative virulence	Mouse	*Rhizopus* spp.	pulmonary	[38,39,48,53,74]
			i.v.	[74,75,76,77]
		*Mucor* spp.	i.v.	[61,72,73,93,99,107,108]
		*Lichtheimia* spp.	pulmonary	[35]
	Rabbit	*Rhizopus* spp.	pulmonary	[62]
		*Mucor* spp.	pulmonary	[62]
		*Cunninghamella* spp.	pulmonary	[62]
		*Lichtheimia* spp.	pulmonary	[65,66]
Therapy and vaccines	Mouse	*Rhizopus* spp.	pulmonary	[39,41,42,43,44,45,46,47,49,51,52,54,56,57,58,59,60]
			i.v.	[36,71,75,94,95,97,101,102,103,104,105]
			cutaneous	[83]
		*Mucor* spp.	i.v.	[43,96,97]
		*Rhizomucor* spp.	i.v.	[97]
		*Lichtheimia* spp.	i.v.	[94,97,113]
		*Cunninghamella* spp.	i.v.	[97,98]
Diagnostics	Mouse	*Rhizopus* spp.	pulmonary	[40,50]
			i.v.	[114]
		*Mucor* spp.	pulmonary	[40]
			i.v.	[114]
		*Rhizomucor* spp.	i.v.	[114]
		*Lichtheimia* spp.	pulmonary	[40]
			i.v.	[114]
		*Cunninghamella* spp.	pulmonary	[40]
	Rabbit	*Rhizopus* spp.	pulmonary	[37]
		*Mucor* spp.	pulmonary	[37]
		*Lichtheimia* spp.	pulmonary	[37]
		*Cunninghamella* spp.	pulmonary	[37]

^1^ Only studies published after 2000 and available online were included. A comprehensive list of studies using mammalian species published before 2000 is presented in Reference [26].

**Table 4:** The advantages and disadvantages of different animal models of mucormycosis.

Feature	Mouse	Rabbit	Galleria	Drosophila
Pathological alterations resemble human mucormycosis	yes	yes	limited	limited
Induction of risk factors in human patients	yes	yes	no	(yes)
Different routes of infection to mimic different types of mucormycosis	yes	yes	no	(yes)
Temperature 37 °C	yes	yes	yes	no
Genetically tractable	yes	no	no	yes
Molecular tools for host response analysis	yes	limited	limited	yes
Costs	moderate	high	low	low
Specialized equipment and personal necessary	yes	yes	no	(yes)
Ethical considerations	yes	yes	no	no
Large-scale screening of fungal strains of antifungal compounds	no	no	yes	yes
